# A home-video method to assess infant gross motor development: parent perspectives on feasibility

**DOI:** 10.1186/s12887-019-1779-x

**Published:** 2019-10-29

**Authors:** M. Boonzaaijer, F. van Wesel, J. Nuysink, M. J. M. Volman, M. J. Jongmans

**Affiliations:** 1 Research Group Lifestyle and Health, Institute of Human Movement Studies, HU University of Applied Sciences, PO Box 12011, 3501 AA Utrecht, The Netherlands; 2 Department of Methodology & Statistics, Utrecht University, Utrecht, The Netherlands; 3 Faculty of Social and Behavioral Sciences, Department of Pedagogical and Educational Sciences, Utrecht University, Utrecht, The Netherlands

**Keywords:** Parental experiences, Feasibility, Home-video method, Infant motor development

## Abstract

**Background:**

Current use of smartphone cameras by parents create opportunities for longitudinal home-video-assessments to monitor infant development. We developed and validated a home-video method for parents, enabling Pediatric Physical Therapists to assess infants’ gross motor development with the Alberta Infant Motor Scale (AIMS). The objective of the present study was to investigate the feasibility of this home-video method from the parents’ perspective.

**Methods:**

Parents of 59 typically developing infants (0–19 months) were recruited, 45 parents participated in the study. Information about dropout was collected. A sequential mixed methods design was used to examine feasibility, including questionnaires and semi-structured interviews. While the questionnaires inquired after the practical feasibility of the home-video method, the interviews also allowed parents to comment on their feelings and thoughts using the home-video method.

**Results:**

Of 45 participating parents, 34 parents returned both questionnaires and eight parents agreed to an interview. Parent reported effort by the infants was very low: the home-video method is perceived as similar to the normal routine of playing. The parental effort level was acceptable. The main constraint parents reported was time planning. Parents noted it was sometimes difficult to find the right moment to record the infant’s motor behavior, that is, when parents were both at home and their baby was in the appropriate state. Technical problems with the web portal, reported by 28% of the parents were also experienced as a constraint. Positive factors mentioned by parents were: the belief that the home videos are valuable for family use, receiving feedback from a professional, the moments of one-on-one attention and interaction with their babies. Moreover, the process of recording the home videos resulted in an increased parental awareness of, and insight into, the gross motor development of their infant.

**Conclusion:**

The AIMS home-video method is feasible for parents of typically developing children. Most constraints are of a practical nature that can be addressed in future applications. Future research is needed to show whether the home-video method is also applicable for parents with an infant at risk of motor development problems.

## Background

In recent years, the necessity of multiple testing to monitor infant motor development adequately has been stated in several studies [1–4]. The use of home videos made by parents could be a way to fulfill this need as it reduces the overall burden of traditional testing on infants and parents. The availability of the Internet and digital cameras, important conditions, seem to have been met, for 98.7% of persons between 25 and 45 years use a smartphone in the Netherlands (Statline, 2018) [5].

**Fig. 1:**
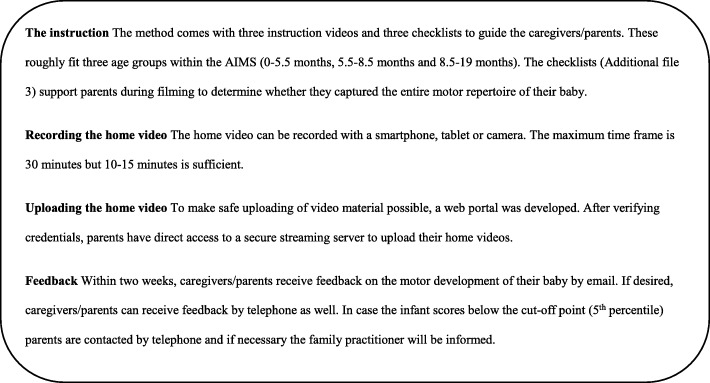
The AIMS home-video method

For this reason, we developed and validated a home-video method which enables professionals to evaluate gross motor performance with the Alberta Infant Motor Scale (AIMS) [6], a valid and reliable assessment tool for infants (0–19 months) [7–10]. An important advantage of this assessment tool is that it evaluates spontaneous motor behavior and requires minimal handling. The home-video method allows parents to record their child’s motor behavior at home and at a convenient time, which increases the chance that the infant will show optimal motor performance [6]. Parents make a home video of their baby, guided by instructions (Additional file 3). Then, they can upload the videos from their smartphone or camera through a computer to a web application which was specifically designed for this purpose. The videos are stored after encryption, with individual encryption keys assigned to each participant. The server has been tested successfully with a high-level security scan by both the institutional security office and an independent outside security office. A Pediatric Physical Therapist (PPT) can then observe the videos and assess the infants’ gross motor development with the AIMS. Unlike a visit to an outpatient clinic, time and geographical distance are no longer barriers [9]. Figure 1 provides a detailed description of the home-video method.

Lately, the use of home videos made by parents to assess or evaluate development has been the subject of several studies [11–15]. Libertus et al. successfully used Skype and FaceTime to assess infants’ early motor skills [13]. Using this method, the digital live connection with parents provided the opportunity to guide parents during the assessment. Although the study stated that using parents in the role of experimenter could lead to increased assessment variability, overall the conclusions on the feasibility for parents were positive. A pilot study by Ricci et al. on the feasibility of filming the General Movements Assessment (GMA is a 3-min video of the infant’s spontaneous movements in supine position) by parents at home after Neonatal Intensive Care Unit discharge showed a less positive outcome [14]. During this pilot, parents experienced major problems recording and sending accurate videos. Therefore, the clinical feasibility of providing adequate home videos made by parents could not be determined. Recently, Spittle et al. launched the Baby Moves Application for parents to record GMA [12]. The usability of the app and the engagement of 451 parents was evaluated by Kwong [15]. This population-based study included 226 infants born extremely premature or with an extremely low birthweight and a control group of 225 term born infants. Overall, positive results on the usability of the application are reported, most parents were able to successfully capture their infant’s movements with the app. All studies carried out so far focus on the practical feasibility of the use of home videos in assessments.

The uniqueness of the AIMS home-video method lies in the fact that parents have a leading role in executing the first part of the assessment, capturing gross motor performance. Apart from the instructions, parents do this on their own. Because most e-Health innovations do not make it to implementation in clinical practice [16], the feasibility of the home-video method for parents needs to be considered carefully [17, 18]. It is important to gain insight into (1) how parents evaluate the practical aspects of the home-video method, and (2) the new role they have in the assessment [17, 19]. Examining these aspects with parents of typically-developing (TD) infants is a first step in our ongoing research project. Parents of infants at risk, using the home-video method, are the ultimate target population.

Thus, the overall objective of this study was to evaluate the feasibility of the AIMS home-video method for parents of TD infants, born at full term and between the ages of 1.5 to 19 months, from the parents’ perspective. In this study, feasibility was defined according to Karsh as ‘the extent to which an innovation can be successfully used or carried out within a given setting’ [18]. According to this construct, we formulated two research questions: (1) how do parents evaluate the practical aspects of the home-video method? and (2) how do parents feel and what do they think about this new method of assessment?

## Methods

### Study design

**Fig. 2:**
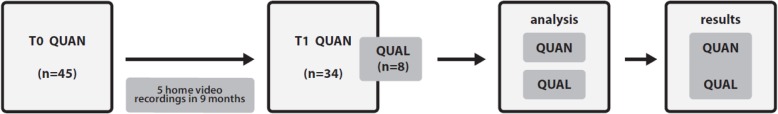
Model of mixed methods design

Because the present study not only focused on the process of the recording but also on parents’ experiences in this specific context, a prospective mixed methods design was chosen [20]. In a mixed methods design, both numeric data and textual information are used, which can be gathered simultaneously or in a sequential manner [20–22]. In the present study, a sequential design was used because of the longitudinal nature of the pilot study [23] (Fig. 2). To evaluate the practical aspects of feasibility, questionnaires were used [18, 19, 24]. To gather more in-depth information on how parents evaluated their new role and to reveal barriers and positive factors, both open-ended questions in the questionnaires and semi-structured interviews were used to collect qualitative data. The quantitative and qualitative data were analyzed separately, and results were integrated while interpreting the findings.

### Setting and participants

Study participants were parents of full-term-born TD infants (1.5–16.5 months) who had participated in a pilot study on longitudinal gross motor trajectories (*n* = 45) in the Netherlands. Parents were instructed to make five home videos of their child with a two-month interval between each video. Two cohorts of infants were included in the study, starting simultaneously. The first cohort comprised 18 infants who started at the age of 1.5 months and were subsequently recorded on video at 3.5, 5.5, 7.5 and 9.5 months. Infants in the second cohort (*n* = 27) were recorded by parents at the ages of 8.5, 10.5, 12.5, 14.5 and 16.5 months. The time frame for making each video was set at exactly 2 weeks. During the study, parents received reminders by e-mail of when to record a video.

The recruitment of parents took place by word of mouth, at social media, day care centers and well-baby clinics by convenience sampling from June 2015 to July 2016. Because of the digital nature, there were no geographical boundaries to participation. Parents expecting or having a full-term-born TD infant and who understood the Dutch language were eligible to enter the study. A subset of eight parents from the study sample was selected for interviewing through a purposive sampling approach to ensure variation in parental and child characteristics, namely age, sex and education level of the parent, birth rank and motor development of the infant. The aim was not to generalize but to obtain a wide view on parental experiences regarding the home-video method.

### Questionnaires and interviews

Online questionnaires were used to enquire into parents’ expectations (T0, before the first video moment) and actual participation (T1, after the last video moment, see Fig. 2) regarding the home-video method. The questionnaires, developed by the researchers, consisted of 21 questions at T0 and 24 questions at T1 (Additional file 1). Questions were included on parent and child characteristics, and on the usability of the home-video method and the web portal. A 5-point Likert’s scale was used (1 = strongly agree it is easy to perform; 2 = agree it is easy to perform; 3 = neutral; 4 = disagree it is easy to perform; 5 = strongly disagree it is easy to perform). A priori, acceptable outcomes in terms of feasibility were set at < 3.

To quantify the expected and experienced effort level for parent and infant (parent-reported), a 10-point scale was used at T0 and T1 (0 = no effort; 10 = a lot of effort).

To obtain information on the children’s longitudinal motor trajectories, Question 21 (T0) and Questions 20–23 (T1) were added to the questionnaires but not included in the current analyses.

A topic list (Additional file 2) provided the basis for the semi-structured interviews. The interviews with the parents, conducted by the first author, took place at home and lasted 30 to 45 min. One respondent preferred to do the interview at work. The interviews were planned after the parent filled out the second questionnaire (T1), recorded on audiotape and transcribed verbatim.

### Ethical aspects

The study was approved by the Medical Ethical Board of the University Medical Centre Utrecht (METC/UMCU) reference nr.14–399/C, and both parents gave written informed consent. Additional written consent was obtained for the interviews.

### Data analysis

#### Quantitative analysis

The mean and standard deviation on single items of the questionnaires (T0 and T1) were calculated. Paired samples *t*-tests and Wilcoxon signed-rank tests were applied to detect changes in expectations and experiences of parents between T0 and T1. Only parents who filled in both questionnaires were included in the analyses (*n* = 34). Statistical analysis was carried out with IBM Statistical Package for the Social Sciences 21.0 (IBM SPSS Statistics for Windows, Version 21.0 Armonk, NY USA).

#### Qualitative analysis

To analyze the data from the interviews and from open-ended questions in the questionnaires, a thematic analysis with a general approach was used, guided by the research questions [25]. After familiarization with the data by reading the transcripts, relevant fragments were coded independently by two researchers (CdB, MB) using MaxQda 10 software [26]. Codings were discussed until consensus was reached. During this process, the codes were categorized into a structured code tree. Emerging themes were identified by constant comparison of codes and text fragments [27]. Although the main focus of the analysis was deductive, based on the topic list, in each phase there was room for inductive elements [28]. The main themes and subthemes that were identified were linked if possible and an overarching interpretation achieved.

## Results

Although 59 parents provided informed consent, 45 participated in the pilot study. Parents who did not send in home videos were approached by telephone to inquire about the reasons for not participating. Reasons for dropping out were: 1) the baby was unexpectedly born prematurely or pathology became evident shortly after birth (*n* = 2); 2) parents reported that in retrospect they were too busy to participate (*n* = 11); 3) frequency of filming was too high (n = 1). Participating parents were residents of 8 of the 13 different provinces in the Netherlands. In total, 45 questionnaires were returned before the start of the study. Following the period of recording the five home videos, 34 surveys were returned (T1; response rate 76%). Table 1 shows the characteristics of participating parents at T0. From this group, 10 parents were approached for an interview. In two cases, parents were unable to schedule an appointment in the allocated period; the other eight parents agreed to an interview.

**Table 1:** Infant, parent and home video characteristics

Infants (n = 45)	
Female (%)	44
Gestational Age in weeks (M, SD)	39.27 (1.45)
Birthweight in grams (M, SD)	3432.7 (504.1)
Birth rank (%)	1st (64) 2nd (30) 3rd (6)
Parents (n = 45)	
Mother/Father (%)	42 (93)/3 (7)
Age (yr, %)	25–30 (24) 31–35 (56) 36–40 (13) 41–45 (7)
Education (%)	Medium (7) High (93)
Home videos	
Total number of recordings Number of recordings per infant (Mdn, Range)	185 4 (1–5)
Device used (%)	Smartphone (60.6) Digital camera (27.3) Tablet (6.1) Other (6.0)

Legend: *M* mean, *SD* standard deviation, *Mdn* median

After analyzing both quantitative and qualitative outcomes, the final thematic framework comprised two main themes: 1) feasibility of the home-video method, in which we combined both quantitative and qualitative data to gain insight into the extent that parents can carry out the home-video method successfully, and 2) parents’ feelings and thoughts that accompany the use of the home-video method. These results were mainly inductive qualitative outcomes.

**Fig. 3:**
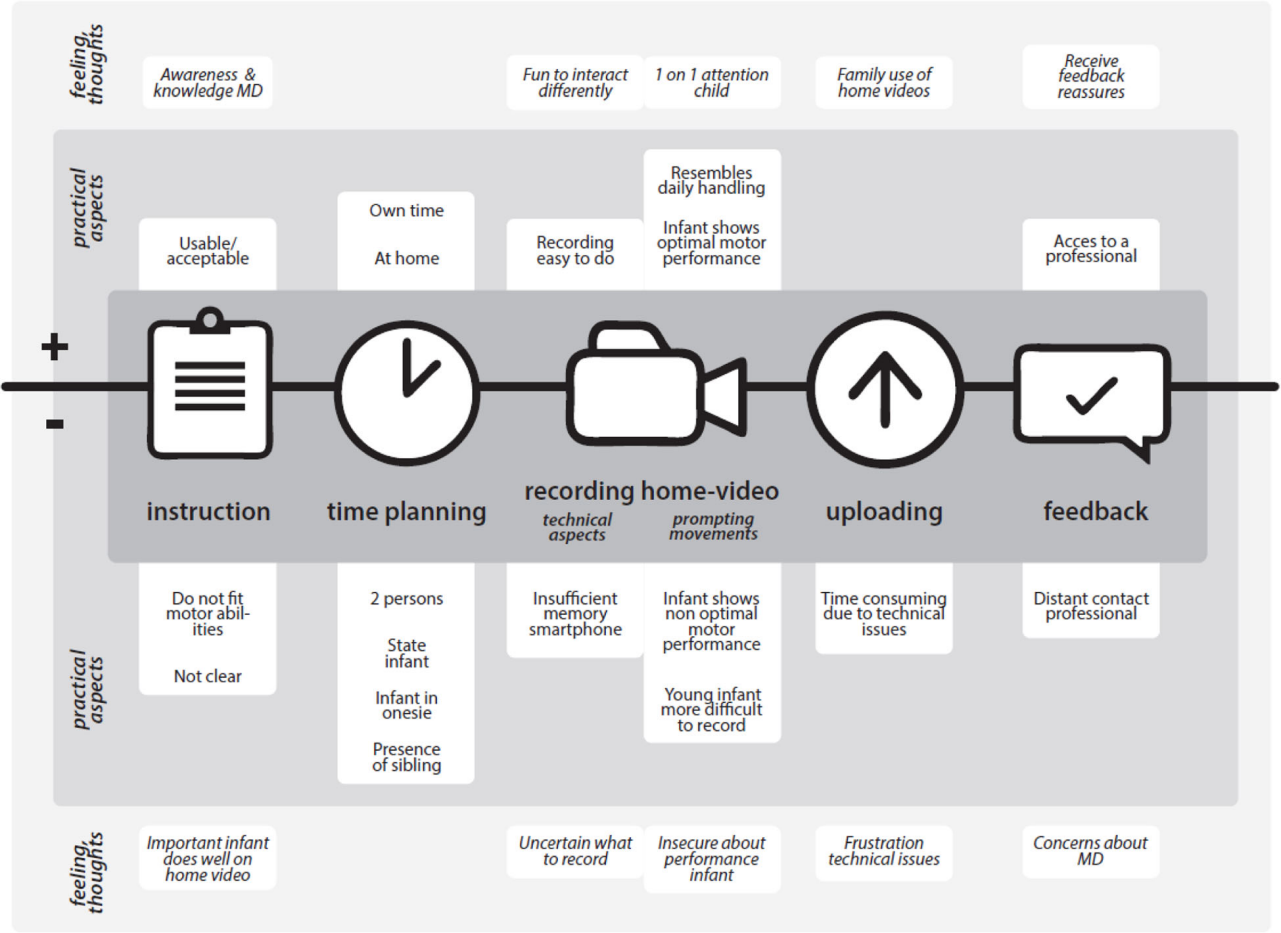
Qualitative results of parents’ perspectives on the feasibility of the AIMS home-video method regarding 1) the practical aspects and 2) the feelings and thoughts

The findings are structured according to the process of making the home video: reading the instructions, planning when to make the recording, recording the home video, uploading the home video, and receiving feedback. First, the quantitative data are presented; next, the qualitative data are used to set the context and to clarify the quantitative findings. In Table 2, the quantitative outcomes are shown and in Fig. 3 the qualitative findings are summarized and visualized.

**Table 2:** Quantitative results of Expectations (T0) and Experiences (T1) of parents applying the home-video method

	T0 Expectations (n = 34)	T1 Experiences (n = 34)	T0-T1
Effort of home-video method (0 = no effort, 10 = a lot of effort)	*M (SD)*	*M (SD)*	Paired t-test (t (df), p)
Parental effort	3.72 (1.67)	4.00 (2.33)	t(33) = − 0.545, *p* = 0.590
Infant effort (parent-reported)	1.97 (1.74)	1.55 (1.48)	t(33) = 1.046, *p* = 0.304
Practical aspects of the home-video method (1 = strongly agree easy to perform, 5 = strongly disagree easy to perform)	*M (SD)*	*M (SD)*	*Wilcoxon’s RT (z, p)*
Technical aspects of recording	1.83 (0.54)	2.10 (0.86)	*z* = − 1,99, *p* = 0.046
Positioning the infant	1.72 (0.53)	1.69 (0.60)	*z* = − 0.26, *p* = 0.796
Prompting movements	2.04 (0.64)	2.07 (0.81)	*z* = − 0.23, *p* = 0.819
Uploading	2.0 (0.89)	3.38 (1.18)	*z* = − 4.08, *p* < 0.001
Finding a convenient moment		3.21 (1.01)	
A 2-week window is sufficient		2.47 (1.05)	
Instruction videos were clear		2.06 (0.74)	
Checklists were clear		1.56 (0.61)	
Feedback no reason for concern^a^		1.93 (1.26)	

Legend: *M* mean, *SD* standard deviation, *df* degrees of freedom, *t* t-value paired samples t-test, *z* z-value Wilcoxon SRT; ^a^outcome item ‘Feedback no reason for concern’ was recoded

### Feasibility of the home-video method according to parents: practical aspects

#### Expected and experienced effort for parents and infants

The quantitative data showed that the expected and experienced efforts of parents applying the home-video method were similar. Qualitative data revealed that parents who appraised the effort higher than expected primarily attributed this to technical issues during the uploading: *‘I didn’t think recording the video was very burdensome. Besides, it was fun to do. But, because of the technical issues uploading the video, it took much longer than expected and that made it somewhat frustrating’ (123, mother).*

Both the expected (M = 1.97, SD = 1.74) and experienced (M = 1.55, SD = 1.48) parent-reported effort of the home-video method for the infant were rated low, and not statistically different for T0 and T1. Parents highlighted this by stating they were primarily recording their baby’s spontaneous movements: *‘The video and the small exercises were no effort for him, I think he actually enjoyed it’ (118, mother).* In some cases, the infant was not in the right state, which made the recording a bit more demanding: *‘For as far I could see, it was no burden on my daughter. Sometimes, she was not in the mood but the exercises were not annoying. Besides, most of the time we were recording her spontaneous movements’ (104, mother).*

#### Instructions

The parents rated the usability of the instructional videos as good. Furthermore, they described the checklists as very usable and clear (M = 1.56, SD = 0.61) (Table 2). The qualitative data supported these findings. Most parents reported viewing the instruction video prior to the recording and using the checklist during the recording: ‘*The checklist was very handy, we had that at hand every time to see: did she show just about everything? It was sort of a guidebook. O.K., we put her down and we have to make sure she does all these items. I also thought, in terms of design, it looked really clear and gave explicit instructions’* (*145, mother*). In a few cases, parents encountered some difficulties applying the checklists because they felt that none of the checklists fitted their infants’ motor abilities adequately at that time: ‘*The first checklist, well, I felt like: this is too easy, he can do all this already. Checklist 1 was far too simple and he couldn’t do much of checklist 2’ (118, mother).*

#### Time planning

Quantitative data showed that parents thought planning the time to record the home video to be an impediment (M = 3.21, SD = 1.01). The 2-week window in which parents could record the video was not always sufficient (M = 2.47, SD = 1.05). These outcomes correspond with the qualitative data where time planning was expressed by a majority of parents as being the main barrier to recording the home video. Parents also mentioned other factors which interplayed with this main barrier. The necessary presence of two persons to record the home video made time planning more complicated. *‘I found it quite hard because we both spent a lot of time at home with her, but not much time with all three of us’ (118, mother)*. One mother explained how the recording of a very young infant could also lead to planning problems: *‘I also breastfeed and certainly in the beginning that takes such a long time so then it’s often when they’re awake you are busy feeding and afterwards they need a change, and those kind of things. Putting them on their tummy and exercising them was not an immediate priority’ (144, mother of twins).*

Also, the fact that parents preferred to choose a moment when the infant was in the right state for recording added up to quite a complex puzzle in today’s dynamic family life. *‘Sometimes it was just difficult timing, you think oh yes now, but then they are tired and then, you really want them to show their best, and then you think: no, they are too tired to do it now’ (114, mother).*

Finally, the presence of an older brother or sister in the toddler or preschool age, could pose a dilemma: ‘*Besides, we have another 5-year-old daughter who we didn’t want to have around at that moment because she wants the attention as well. We really needed to look for occasions when she wasn’t at home’ (124, mother).*

Parents also experienced favorable aspects of the home-video method, such as being able to video the infant at home in their own time without a professional coming over to assess the infant’s gross motor development. ‘*Would I have preferred a researcher coming over here for each video moment? On the one hand, then you make the appointment and then it is set, yes? But then you’re stuck with it. This way, I could plan it in my own time. So, that’s a big advantage of doing the recording by yourself’ (136, mother).*

A father puts it like this: ‘*It is of course very accessible, you don’t have to leave, nobody has to come to your house and you can record a video and get a reaction to that. So I think it can only be more convenient’ (152, father).*

The home appeared not to be the only suitable place for recording the videos. In multiple cases, infants were recorded during a visit or stay with the grandparents. Also, during holiday seasons, some parents sent home-video material from camping sites, apartments and cottages from all parts of the world. *‘We went on a holiday and made the videos, and once we recorded the video at my parents’ house, so we did film her at diverse locations. With such a small baby that is no problem, of course.’ (136, mother).*

#### Technical aspects of recording

In advance (T0), parents did not expect (M = 1.83, SD = 0.54) the technical aspects of the recording (i.e. camera position, light and distance) to become a problem. At T1 (M = 2.0, SD = 0.86), the experience was rated not much but still statistically significantly higher in difficulty (Z = − 1.99, *p* = 0.046) (Table 2). The opinion of most parents can be gathered under this parent’s expression: *‘The recording itself was not hard to do; I do it every day!’(141, mother).* However, due to the daily use of the smartphone as a camera, some parents already had a lot of photo and video files stored on their smartphone. This might explain the significant negative change in the experiences parents had regarding the technical aspects: ‘*After a few videos, the memory card in my smartphone was full. So I had to upload and remove photos, which takes time. After that I’m able to continue recording, in the hope my baby still wants to cooperate’ (114, mother)*.

#### Positioning the infant and prompting the movements

Parents found it easy to position their child in accordance with the instructions (M = 1.72, SD = 0.53 expected and M = 1.69, SD = 0.60 experienced: Table 2). This can be understood from the qualitative data too, where parents explained that it mostly resembles daily handling: *‘She did what she is always doing, only now with a bit more facilitation and a camera present’ (152, father).*

Parents also rated the prompting of specific movements as feasible to perform (M = 2.04, SD = 0.64 expected and M = 2.07, SD = 0.81 experienced: Table 2). A mother expressed in the interviews: *‘You really prompt her, yes. She has now reached out with her right arm and then you try to get her to reach with her left arm also. So that’s what I really enjoyed’ (145, mother).*

Although most infants were recorded at a convenient time and in the right state, some parents noted that their infant did not show optimal motor performance during recording. In the questionnaire, 23% of parents indicated that their child did not show optimal motor performance in the final home video. Reasons for this were 1) the state of the infant, 2) the infant was distracted by the camera and 3) by coincidence. This could lead to some frustration for both parent and child: ‘*It was hard to find a moment he was in the right mood … so sometimes he got frustrated for not showing things he normally would show and we were waiting for him to show that behavior’ (123, mother)*. However, 77% of parents stated their child did show optimal motor performance or even showed new motor abilities during the recording.

#### Uploading the home video

In advance, parents did not expect that uploading the home videos to the web portal would lead to any obstacles (M = 2.00, SD = 0.89). However, afterwards this theme demonstrated a significant negative change (M = 3.38, SD = 1.18, *p* < 0.001). Due to instability of the software during the pilot, the web portal was not always functioning properly which made uploading more time-consuming. Approximately, 28% of parents encountered these difficulties. Parents also reported this as a factor that increased the overall effort they experienced during the pilot. Where mothers were most involved in the study, fathers played an important role in dealing with the digital problems. ‘*I kept aloof from that* [uploading home videos]*, I am not that into transferring videos onto the computer, so that was my husband’s thing. I was into the recording and telling him what we had to do and he mainly did the technical part’ (136, mother).*

#### Receiving feedback

In the questionnaires, most parents reported that the feedback on the motor development of their child gave no cause for concern (M = 1.93, SD = 1.26) (Table 2). Furthermore, some parents reported that the feedback and access to an expert on motor development they could turn to with questions was an agreeable aspect of participating*. ‘And if something goes wrong, he lags behind or there is a handicap, that you know it in time. That there are professionals monitoring your baby who can intervene in time. So you don’t just find out at the age of 4 that he can’t throw a ball’ (114, mother).* In this context, the feedback was mentioned as an important motivator to stay involved in the study.

One parent thought the feedback was a less important part of the process. For her, seeing her baby perform was the most enjoyable element: *‘The feedback was nice to see but the fun part was the moment that you record her and see her doing it‘(145, mother*).

### Parental perspective on the new role: feelings and thoughts

In addition to the perspective parents gave on the practical aspects of feasibility, they also expressed their ‘feelings and thoughts’ which accompanied their new role in applying the AIMS home-video method. Parents expressed both negative and positive feelings and thoughts. In Fig. 3, these results are displayed in the outer part of the model.

During recording, some parents experienced insecurity about the motor development of their child. Also, some of them reported insecurities about whether they had recorded the movements and postures as intended. Especially when recording for the first time, they expressed questions about the duration of the recording and how long they should keep on facilitating: *‘You are just not sure if you did the recording the right way, so I just went ahead and made the video but still I wasn’t certain’ (118, mother).*

The qualitative data showed that a few parents, whose children scored below average on one or more occasion, did experience some concerns when they received feedback: ‘*At the start, I found it a bit difficult to see that T. scored quite low, but that was a result of my insecurity as a mother’ (106, mother).*

Almost all parents expressed it was important their child would show the best on the home video: ‘*At that moment, I wanted him to show the good things, yes I felt quite strong about that. After all, you would get feedback on it and it was about his development. You knew he already was able to do some things but when he was tired, he didn’t show it that well*’ *(114, mother).* Some parents even considered making a new recording because they were not satisfied with the first. However, parents refrained from this because of time constraints.

Many parents reported that, despite the effort involved, they did enjoy the individual attention and time spent with their baby: ‘*And somehow, with your firstborn you probably have it* [one-on-one attention] *more. She is my second and I almost felt like I wanted to give her this attention to her motor development’ (145, mother).*

The active involvement of parents in recording the home video appeared to have some side effects triggered by the fact that parents interacted with their baby in a different way. By looking at the instruction video and the checklists, several parents reported they gained knowledge about, and became more aware of, their baby’s motor development: *‘So I did notice, especially in the beginning, that suddenly you start realizing what she is doing. You really start very focused observing’ (145, mother).* In one case, parents were alarmed by what they observed in the instruction videos: ‘*By looking at the instruction videos, we realized that our son lagged behind in his motor development, so we contacted a Pediatric Physical Therapist’ (121, mother).*

Some parents also acquired new insights in how to optimize motor development: ‘*Yes, well also regarding tummy time, we found out that the baby enjoyed to move around on a larger surface. Because we saw the effect it had, we did it more often’ (114, mother).*

For the participating parents, who all have TD infants, the main encouragement to participate was to obtain valuable home video material which captured the motor development of their baby over a period of time. Another key to compliance was the feedback on their infants’ motor development. Parents found the extra developmental monitoring of their infant both reassuring and interesting.

## Discussion

The present study explored the experiences of parents in using a home-video method to assess their infants’ gross motor development. Overall, parents were positive about the practical feasibility of the home-video method. They reported that the recordings were easy to do and that the handling of the baby was mostly as in daily routines. Several barriers were identified in this study. The main barrier reported was time planning. A second barrier concerned technical problems with the web portal, which sometimes made uploading the home-videos time consuming. According to parents, positive factors of this home-video method were (1) that the home videos were valuable for family use, (2) that receiving feedback from a professional about infants’ motor development was welcome, and (3) that it was fun to interact with their babies in a different way and to have a moment of one-on-one-attention. Moreover, the instructions and home video recording resulted in an increased parental awareness of, and insight into, the gross motor development of their infants. The feelings and thoughts parents expressed about their new role were both positive and negative. In some cases, parents expressed their uncertainty about the motor performance of their child or about the video recordings. Parents also reported joyful feelings about the interaction they had with their baby while making the home videos. In addition, most parents appreciated the feedback on the motor development of their child which they found reassuring.

For future application, it is important to address all barriers identified in this study [19]. Time planning is mentioned most explicitly: parents were hard pressed to find a moment when they both were at home and their baby was in the proper state to show optimal motor behavior. During the study, some parents found a solution to the logistics: by positioning their phone on the table or floor, they managed to record and handle their infant at the same time.

From the results, we can conclude that a functional and user-friendly digital application is an absolute prerequisite for successful implementation of this method. This is exactly in line with the conclusions of Ricci et al. [14]. The main barrier they described was the use of an encrypted server with very high protection levels, obligated because the home videos were considered to be personal health information. In this study, the server was security tested and found to be compliant to relevant laws (NEN 7510/7512/7513 norms). The encryption of data while uploading is important to ensure safety but as a consequence the uploading was sometimes time consuming. This was also the case for the assessors while downloading and decrypting the video data. Both aspects limit feasibility and should be addressed. A satisfactory compromise between functionality and safety in the development of health care applications seems an important step towards successful implementation in practice.

In addition, in the development and use of digital communication means, the privacy of parents and infants is considered to be very important [29]. In our study, privacy issues did not emerge as a significant theme. Perhaps digital privacy is not an important issue for all parents. Ricci et al. [14] reported that, because of the problems uploading the videos, many parents offered to share the home videos on open platforms like Facebook or WhatsApp. In our study we had similar experiences. This might also be in line with the findings of Hassol and colleagues, who reported that only a minority of patients was concerned about the privacy of their electronic health care record [30]. However, a self-selection bias may have occurred in the privacy aspect. Parents with explicit ideas on privacy regarding video material of their child may have decided not to participate in the present study from the start.

Libertus and Violi, who used Skype as a means of collecting developmental data, suggested that access to the Internet and digital equipment could also be a constraint for parents’ participation in these kind of research projects [13]. In our homogeneous sample, all parents had access to the Internet and a smartphone. According to Statistics Netherlands, over 98% of persons aged 25–45 years have access to the Internet and almost 95% own a smartphone [5]. These high percentages lead us to believe this aspect unlikely to be a limiting factor for participation in our study.

Only a few studies describe the feasibility of digital screening methods for parents at home [11, 13–15, 24]. Besides, every method has its own specific features which affect parental experiences and thus feasibility in different ways. The evaluation of the usability of the Baby Moves app shows that most parents successfully used the app to record their baby’s movements [15]. However, because the AIMS home-video method is more demanding for parents, it is questionable whether these results can be applied to the AIMS home-video method. Our positive findings on the feasibility of the AIMS home-video method are more comparable to a study on a video-based evaluation tool for children with Rett syndrome [11]. In this study, outcomes on feasibility were positive, despite the fact that parents had to follow quite extensive instructions to record multiple abilities and interactions. Furthermore, these authors reported benefits from recording the child in a familiar setting. We think this aspect also applies to a large extent to the AIMS home-video method. On most home videos, infants’ state was suitable for testing. When assessing motor development from the recordings, it was seen that the infant didn’t have to adapt to a new environment, strange people or a set appointment time, which is the case when the infant is seen in a PPT practice or hospital outpatient clinic.

Although some parents reported that their child did not always show optimal motor performance on the home videos, we speculate that this might be overstated. The importance parents placed on their child’s showing optimal motor performance on the home video might sometimes have resulted in a more negative perception of the child’s performance. For example, if an infant had shown rolling over from supine to prone for the first time just before the recordings, it is quite likely not to be shown in the home video, and parents could feel disappointed about this. For a professional assessing the home video, not seeing the infant rolling over would not necessary influence the validity of the assessment; rolling over might just not yet be in the infant’s motor repertoire.

An important finding of this study is the teaching effect the AIMS home-video method potentially has. The method requires active parental involvement which can lead to a better understanding of the infant’s motor development [31, 32]. Parents with an infant at risk for delay might especially benefit from this knowledge. It might help them to become ‘their child’s expert’ even more and as such improve equality in shared decision-making between parents and professionals [33].

### Strengths

This study is the first that not only reports outcomes on practical feasibility of home video assessments but also attempts to grasp the feelings and thoughts of parents. Parents are the most important stakeholders in the home-video method and their experiences have to be acknowledged for successful implementation. The mixed methods design, a combination of questionnaires and interviews, provided rich information about the experiences of parents. The main outcomes of both qualitative and quantitative data reinforced each other and were thus complementary. The interviews clarified and illustrated the quantitative findings [22]. The thematic analysis with a combined approach, both deductive and inductive, brought forth important new insights in parents’ feelings and thoughts regarding the home-video method. Another strength concerns the longitudinal nature of the study, which allowed parents to report on multiple experiences with the recording of their child, instead of a one-time exposure. Because of this design, it was also possible to inquire after the expectations of parents before the start of the study.

### Limitations

Our study is subject to the following limitations. The advanced educational level of the majority of participating parents limits the generalizability of the results. The checklists do demand some literacy and might therefore not fit parents who are less educated. On the other hand, the additional instruction videos could partially solve this barrier. In the population-based study of Kwong et al., it became evident that families of lower socio-economic status who used the Baby Moves app were less likely to return scorable videos [15]. Education and socio-economic status are important variables that might also interplay with the feasibility of the AIMS home-video method and need to be addressed in further studies.

The dropout rate in this study was considerable which threatens feasibility. However, we investigated both the feasibility of the home video method and the feasibility of applying it longitudinally. We asked parents with a young baby to commit themselves to the study for a period of 9 months. All parents who participated in the pilot, delivered one to five adequate home videos, which shows the home-video method itself was feasible for these parents. It was mainly the final questionnaire (T1) which was returned poorly (*n* = 34). These data indicate that the longitudinal aspect of the study was probably the main reason for dropout. Another limiting factor was that a majority of parents who signed up to participate (filled out the questionnaires and participated in the interviews) were mothers. The young age of some of the participating infants (as low as 1.5 months at the start) might have played a role in this phenomenon. Having maternity leave, Dutch mothers were probably more available and willing to become involved in research than fathers. Although most parents worked together to record the home videos, it was mainly the experiences of the mothers that were collected in both questionnaires and interviews. This is a known limitation in infant studies [34] and it is important to consider because fathers might have different experiences than mothers, especially with regard to digital equipment.

## Conclusion

The present study provides evidence that the AIMS home-video method is feasible for participating parents regarding both practical aspects and the understanding of their task. Most identified barriers reported by parents have a practical nature that can be addressed in future applications. The home-video method has the potential to become a valuable E-health addition for both research and PPT practice to monitor infants at risk of developmental motor delay in their own familiar environment.

More research is needed to explore if these findings are applicable to parents with different backgrounds and to parents of infants at risk. How will these parents experience a more explicit role in the assessment of their child’s risk for a delay in motor development? Will the active involvement of parents indeed lead to increased awareness and knowledge of motor development? In short, can the AIMS home-video method become more than just a means and become a tool to empower parents who have an infant at risk of developmental delay?

## Supplementary information

**Additional file 1:** Online questionnaire for parents.

**Additional file 2:** Topic list used for interviews with parents.

**Additional file 3:** Checklist I - III AIMS home-video method for parents.

## Data Availability

The datasets used during the current study are available from the corresponding author on reasonable request.

## Abbreviations

AIMS: Alberta infant motor scale
GMA: General Movements Assessment
MD: Motor development
PPT: Pediatric physical therapist
TD: Typically developing
