# Supplementary material for: A home-video method to assess infant gross motor development: parent perspectives on feasibility
Source: BMC Pediatr. 2019 Oct 29;19:392. doi: 10.1186/s12887-019-1779-x (PMC6819354; doi:10.1186/s12887-019-1779-x)
Supplement: Supplementary file 1 — Additional file 1: Online questionnaire for parents. [file 12887_2019_1779_MOESM1_ESM.pdf]

Additional file 1. Digital Questionnaires (T0 and T1)

Questionnaire for parents (T0): Expectations on the home-video method

Personal data

1. Please enter your child's research code.
2. What is the date of birth of your child?
3. What was the duration of pregnancy?
4. What was the birthweight of your child?
5. What is the birth rank of your child?
6. What is the name of the general practitioner?
7. What is the place of residence of the general practitioner?
8. What is your age?
9. What is the age of your partner?
10. What is your level of education?
11. What is your partner's level of education?
12. What is your relationship with the child (father/mother/other)
13. Because of my work/education, my knowledge of infant motor development is more than average

- I strongly disagree
- I disagree
- Neutral
- I agree
- I totally agree

Questions on expectations

14. How much effort do you expect this study will cost you as a parent?
    Give a number from 0-10 (0 = no effort at all, 10 = a lot of effort)

15. How much effort do you expect this study will cost your baby
    Give a number from 0-10 (0 = no effort at all, 10 = a lot of effort)

16. What is your motivation to participate in this study?

- I think the study is useful and interesting.
- I think participation in research project is important.
- It is nice to know about the motor development of my child.
- I have a question on the motor development of my child.
- Other:

17. I expect that the technical aspects of recording the home video (light, distance, camera position) will be easy to carry out.

- I strongly disagree
- I disagree
- Neutral
- I agree
- I totally agree

18. I expect that deciding which positions and movements of my child I have to capture on home video will be easy to do.

- I strongly disagree
- I disagree
- Neutral
- I agree
- I totally agree

19. I expect that prompting my child to show specific movements will be easy to do.

- I strongly disagree
- I disagree
- Neutral
- I agree
- I totally agree

20. Uploading the home videos using the web portal on the computer will not be a problem for me.

- I strongly disagree
- I disagree
- Neutral
- I agree
- I totally agree

21. Can you describe your child's current motor development?

- Faster than average
- Average
- Slower than average
- I have no idea

Questionnaire for parents (T1): Experiences of the home-video method

I Questions on the experiences

1. Please enter your child's research code.

2a. How would you rate the effort of this study for you as a parent?

Give a number from 0-10 (0 = no effort at all, 10 = a lot of effort)

2b. Please explain the given number.

3a. How would you rate the effort of this study for your child?

Give a number from 0-10 (0 = no effort at all, 10 = a lot of effort)

3b. Please explain the given number.

4. It was easy to find an appropriate moment to record the home videos.
   - I strongly disagree
   - I disagree
   - Neutral
   - I agree
   - I totally agree

5. Two weeks' time is enough to find an appropriate moment to record the home-videos.
   - I strongly disagree
   - I disagree
   - Neutral
   - I agree
   - I totally agree

6. The instruction videos were clear and understandable.
   - I strongly disagree
   - I disagree
   - Neutral
   - I agree
   - I totally agree

7. The checklists were clear and understandable.
   - I strongly disagree

- I disagree
- Neutral
- I agree
- I totally agree

8. What device did you use to record the home-videos?
   - Smartphone: iPhone
   - Smartphone : Android
   - Tablet: iPad
   - Tablet : Android
   - Digital camera
   - Other:

9. Uploading the home videos to the web portal from my computer was easy to do.
   - I strongly disagree
   - I disagree
   - Neutral
   - I agree
   - I totally agree

10. Did you experience any difficulties using the device while recording and/or uploading?
    - No
    - Yes, being …

11. To upload the home videos I used:
    - The web portal
    - We Transfer
    - Other:

12. The technical aspects of the recording were easy to carry out

(light, distance, camera position).

- I strongly disagree
- I disagree
- Neutral
- I agree
- I totally agree

13. It was clear for me what positions and movements of my child I had to capture on camera.

- I strongly disagree
- I disagree
- Neutral
- I agree
- I totally agree

14. Prompting my child to show specific movements was easy to do.

- I strongly disagree
- I disagree
- Neutral
- I agree
- I totally agree

15. The feedback I received by email was informative enough for me.

- I strongly disagree
- I disagree
- Neutral

- I agree
- I totally agree

16. The feedback I received on my baby’s motor development gave cause for concern.
    - I strongly disagree
    - I disagree
    - Neutral
    - I agree
    - I totally agree

The following questions only apply to the last time you recorded a home video of your child.

17. How was your baby’s behavioral state during the recording?
    - Sleepy, not active
    - Quiet, less active than normal
    - Cheerful, active
    - Irritable, grumpy
    - Upset, crying
    - Other:

18. On the home video my child showed optimal motor behavior.
    - Yes my child showed his/her optimal motor behavior
    - Yes, and my child even showed new items
    - No, my child did not show optimal motor behavior

      (please answer question 19)

19. Please indicate what factors you think had a negative influence on your child’s motor behavior during the video recording:

- My child was disrupted by the camera
- My child was disrupted by noises or bustle
- My child had some physical inconveniences
- My child was not in the mood/ right state
- The presence of other people disrupted my child
- It was coincidental that my child didn't show optimal motor behavior
- Other:

20. What do you think about your child's current motor development?
    - Faster than average
    - Average
    - Slower than average
    - I have no idea

21. Who has most care tasks at the moment?
    - Mother
    - Father/partner
    - Mother and father/partner have equal caring tasks
    - Other:

22. Can you indicate your baby's residency for the days of an average week?
    (at home, with grandparents, daycare, other)

23. Can you indicate factors that might have influenced your infant's motor development during this study? Please indicate at what age.
    (Illness, treatment by a (para)medic, change in family composition, change in living environment, move to a new house, change in daycare)

24. This questionnaire has been completed by:
    - Father/partner
    - Mother
    - Other:
