# Supplementary material for: A home-video method to assess infant gross motor development: parent perspectives on feasibility
Source: BMC Pediatr. 2019 Oct 29;19:392. doi: 10.1186/s12887-019-1779-x (PMC6819354; doi:10.1186/s12887-019-1779-x)
Supplement: Supplementary file 2 — Additional file 2: Topic list used for interviews with parents. [file 12887_2019_1779_MOESM2_ESM.pdf]

Additional file 2. Semi-structured topic list interview

I The instruction

- Beforehand, was it clear to you what was expected?
- How much time did you need for preparation?
- What did you think about the instruction videos?
- What did you think about the checklists?

II Recording and uploading the home-video

- Please describe how the recording of the home video came about?
- Which digital device did you use to make the recording?
- What did you think about the instructions: were they clear?
- What were your experiences uploading the recordings?

III Handling and prompting the infant

- How did you feel about handling your baby and prompting movements according to the instructions?
- Were you able to find an appropriate time for recording?
- Was it clear to you which postures and movements you were supposed to record?
- Was your baby able to show his/her optimal motor performance during the recording?

IV Feedback on motor development

- What do you think about the feedback you received on the motor development of your baby?
- Did the feedback influence your actions or thoughts towards/about your baby?

V Experiences in general

- How did you experience your participation in this research project in general?
- How was it to make multiple recordings over a time of 9 months?

- What motivated you to stay involved?
- How could we improve parental compliance even more?
- What are your thoughts on the safety of the video recordings of your child and privacy issues?
- Do you think the home-video recording method is feasible for all parents?
- What do you think about the home-video method to assess an infant’s motor development?
- Do you have ideas to improve the home-video method?
- Is there anything you would like to add or comment on?
