# Supplementary material for: A home-video method to assess infant gross motor development: parent perspectives on feasibility
Source: BMC Pediatr. 2019 Oct 29;19:392. doi: 10.1186/s12887-019-1779-x (PMC6819354; doi:10.1186/s12887-019-1779-x)
Supplement: Supplementary file 3 — Additional file 3: Checklist I - III AIMS home-video method for parents. [file 12887_2019_1779_MOESM3_ESM.pdf]

Appendix I Checklist I-III AIMS home-video method

Checklist I The baby is not rolling over yet

This checklist can be used during filming. Don't forget to watch the instruction video.
In this checklist you will find:
- The movements and positions we want you to capture on the home video.
- Tips to pay attention to, so your home video can be used to assess motor performance.

Check	Tips	
General	☐ We will assess the motor skills of your baby, so let him/her move freely and try not to help with your hands.	
	☐ A good way to start the video is to film spontaneous movements of your baby; please don't elicit movements with toys or sounds right away.	
	☐ During filming, make contact with your baby like you always do.	
	☐ The positions we ask you to film do not have to be filmed in the order displayed. Breaks can be taken if that's desirable.	
	☐ If you make the home video with your smartphone, the phone has to be in a horizontal position.	
	☐ During filming, your baby should only be wearing a body suit.	
Environment	☐ Try to film with the light source behind you.	
	☐ Please film your baby on the floor and make sure the under layer is firm and prevents sliding.	
Duration and timing	☐ Please make sure you have 10-15 minutes on tape. The maximum length of the home video is 30 minutes.	
	☐ Try not to make multiple short video shots. We prefer longer shots.	
	☐ When your baby is getting tired or discomforted, it is better to stop and try filming again another time.	

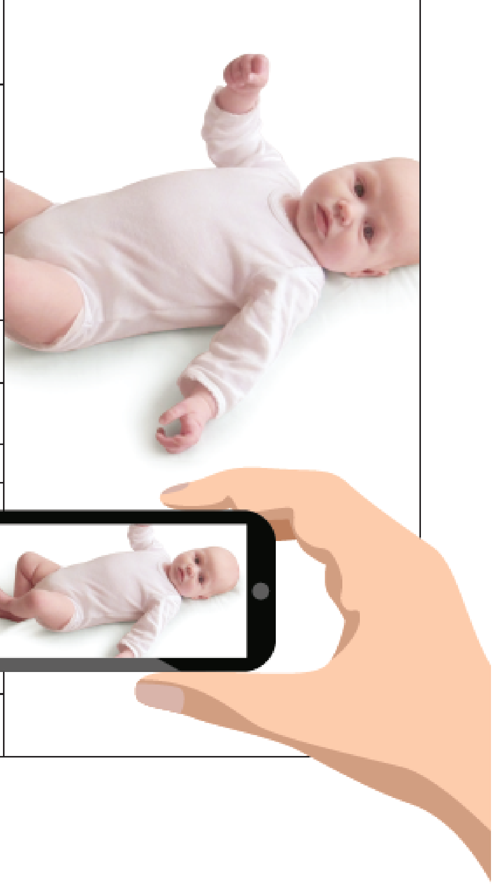

* The development of this checklist was part of a grant research project (2013-53p).

UNIVERSITY OF APPLIED SCIENCES UTRECHT

Checklist I The baby is not rolling over yet

Position	Check the tips	Camera position from the	
Supine	☐ Film a few minutes in supine position without a toy.	side<br>bottom	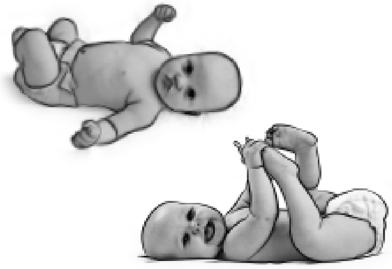
	☐ Present a little toy above your baby, to elicit reaching and/or grabbing.	side<br>bottom	
Prone	☐ Lay your baby down in prone position with his/her hands at shoulder level. Film a few seconds without making contact. After that make contact with your baby to see if he/she is able to actively raise the head.	side<br>top	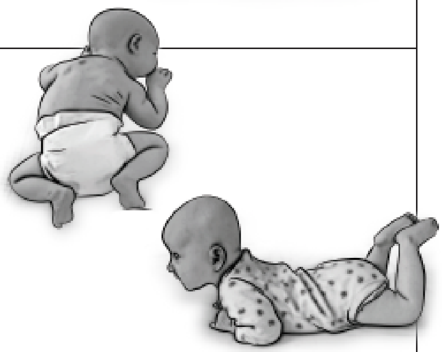
	☐ Present a small toy right in front of your baby.	side<br>top	
Pull to sit	☐ Make eye contact with your baby in supine position, so he/she turns the head to the midline. Then hold the wrists of your baby and pull gently. When the head still lags behind, lay down your baby gently. Repeat this one more time.	side	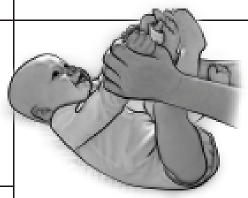
Sitting with support	☐ Keep your baby supported in the sitting position and see if you can make eye contact.	front<br>side	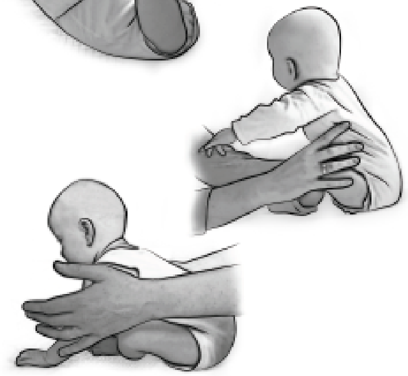
	☐ See if your baby can sit without support for a brief moment. Your baby may use the arms as support forward. Keep your hands close by, sitting is not a stable position yet.	front<br>side	
Supported standing	☐ Hold your baby between the pelvic and the shoulders. Let the feet touch the floor and see if your baby takes some weight on the feet or toes.	front<br>side	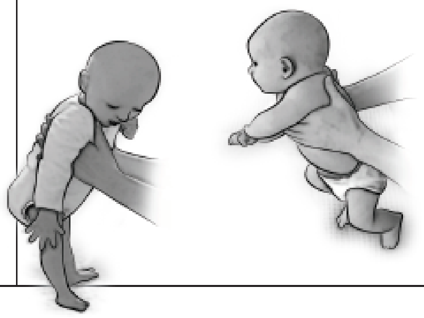

Checklist II The baby is rolling over and starting to move

This checklist can be used during filming. Don't forget to watch the instruction video.
In this checklist you will find:
- The movements and positions we want you to capture on the home video.
- Tips to pay attention to, so your home video can be used to assess motor performance.

Check	Tips	
General	☐ We will assess the motor skills of your baby, so let him/her move freely and try not to help with your hands.	
	☐ A good way to start the video is to film spontaneous movements of your baby; please don't elicit movements with toys or sounds right away.	
	☐ During filming, make contact with your baby like you always do.	
	☐ The positions we ask you to film do not have to be filmed in the order displayed. Breaks can be taken if that's desirable.	
	☐ If you make the home video with your smartphone, the phone has to be in a horizontal position.	
	☐ During filming, your baby should only be wearing a body suit.	
Environment	☐ Try to film with the light source behind you.	
	☐ Please film your baby on the floor and make sure the under layer is firm and prevents sliding.	
Duration and timing	☐ Please make sure you have 10-15 minutes on tape. The maximum length of the home video is 30 minutes.	
	☐ Try not to make multiple short video shots. We prefer longer shots.	
	☐ When your baby is getting tired or discomforted, it is better to stop and try filming again another time.	

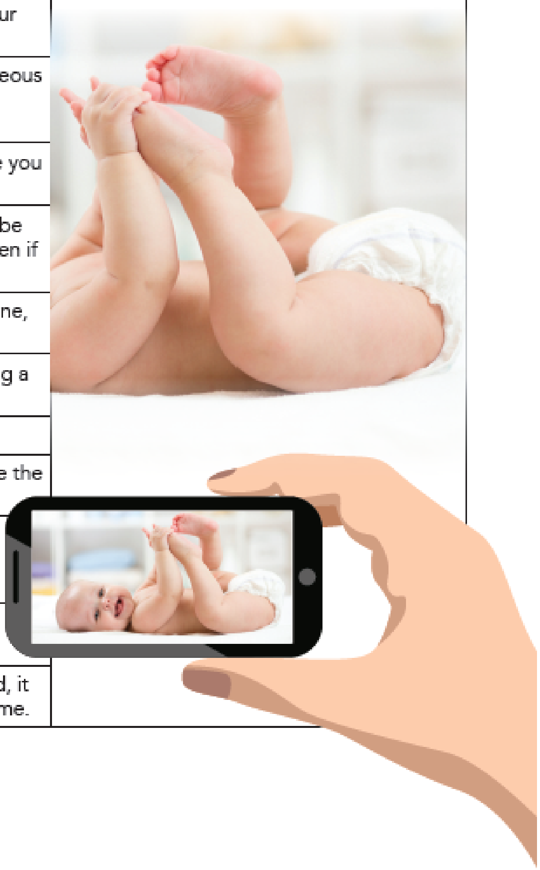

* The development of this checklist was part of a grant research project (2013-53p).

UNIVERSITY OF APPLIED SCIENCES UTRECHT

Checklist II The baby is rolling over and starting to move

Position	Check the tips	Camera position from the	
Supine	☐ Please film a few minutes in supine position without a toy	side<br>bottom	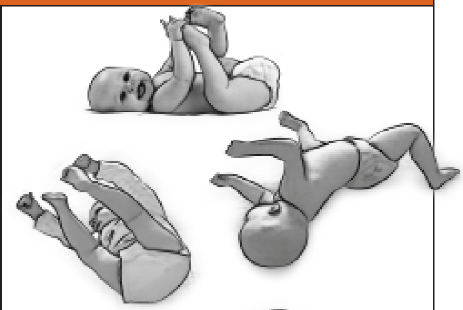
	☐ Present a little toy above your baby, in that way you can elicit reaching and/or grabbing		
	☐ Present a toy beside the head of your baby, maybe he/she will roll over		
Prone	☐ If needed, help your baby to lay down in prone position. Film the spontaneous movements for a short while.	side<br>top	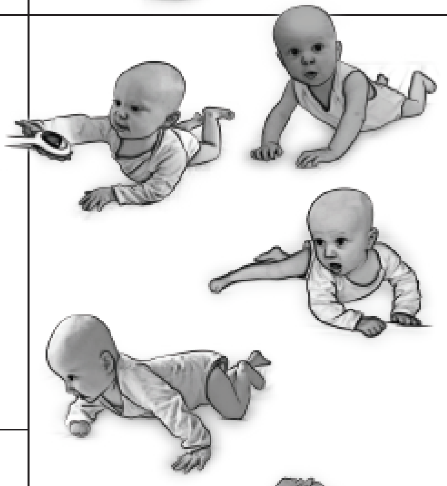
	☐ After that, present a toy in the sight of your baby; in front of him/her.		
	☐ Present a toy above the head and shoulders. Try to elicit reaching or grabbing the toy by leaning on one arm. Try this at both sides.		
	☐ Present a toy and move it in a circle around your baby so he/she will follow it. Now your baby is dialling on his/her belly.		
	☐ If you know your baby can move forward on the belly, try to capture this.	side	
Pull to sit	☐ Hold the wrists of your baby and pull gently to the sitting position. Please film this movement one more time.	side	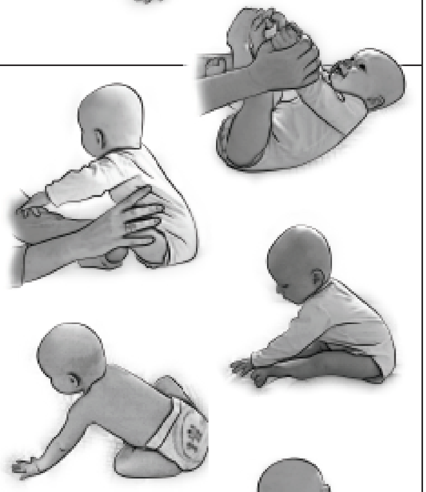
Supported sitting	☐ Keep your baby supported in sitting position and see if you can make eye contact.	front<br>side	
	☐ See if your baby can sit on his/her own for a brief moment.		
	☐ If you know your baby can transfer from sitting to supine position, film this.	side	
Standing	☐ Hold your baby between the pelvic and the shoulders. Let the feet touch the floor to see if he/she takes some weight on the feet.	front<br>side	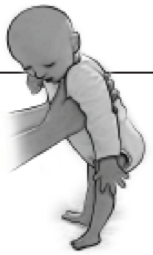

Checklist III The baby is crawling and/or walking

This checklist can be used during filming. Don't forget to watch the instruction video.
In this checklist you will find:
- The movements and positions we want you to capture on the home video.
- Tips to pay attention to, so your home video can be used to assess motor performance.

Check	Tips	
General	☐ We will assess the motor skills of your baby, so let him/her move freely and try not to help with your hands.	
	☐ A good way to start the video is to film spontaneous movements of your baby; please don't elicit movements with toys or sounds right away.	
	☐ During filming, make contact with your baby like you always do.	
	☐ The positions we ask you to film do not have to be filmed in the order displayed. Breaks can be taken if that's desirable.	
	☐ If you make the home video with your smartphone, the phone has to be in a horizontal position.	
	☐ During filming, your baby should only be wearing a body suit.	
Environment	☐ Try to film with the light source behind you.	
	☐ Please film your baby on the floor and make sure the under layer is firm and prevents sliding.	
Duration and timing	☐ Please make sure you have 10-15 minutes on tape. The maximum length of the home video is 30 minutes.	
	☐ Try not to make multiple short video shots. We prefer longer shots.	
	☐ When your baby is getting tired or discomforted [illegible] is better to stop and try filming again another tim[illegible]	

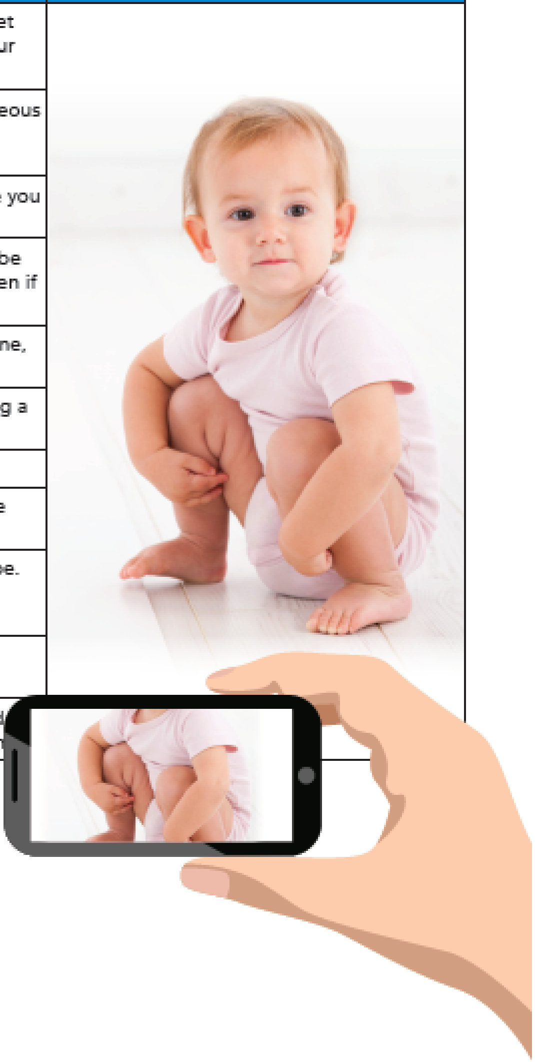

* The development of this checklist was part of a grant research project (2013-53p).

UNIVERSITY OF APPLIED SCIENCES UTRECHT

Checklist III The baby is crawling and / or walking

Position	Check the tips	Camera position from the	
Supine	☐ Please, turn on the camera when you take off your baby's clothes in supine position.	side	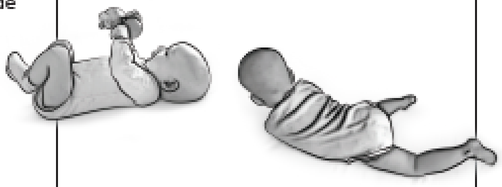
	☐ At this age, your baby can roll over very easily. Try to capture this movement to both sides.		
Prone	☐ Capture your baby moving forward, this can be crawling or creeping.	side	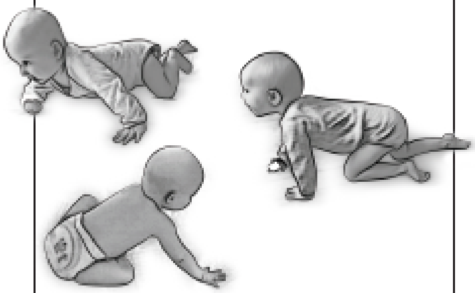
	☐ Try to film the transfer from sit to crawling.		
Sitting	☐ Film your baby while he / she is transferring to sit. Let it play with some toys in this position.	side	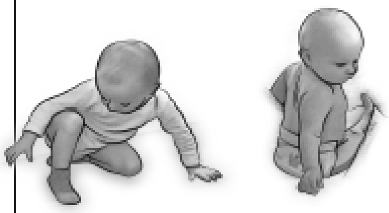
	☐ Present a toy to your baby at both the left and the right side and out of reach, so he / she has to turn to reach for the toy.		
Standing	☐ Put some toys on the couch or the table. If your baby does not pull up to a standing position, help your baby on the feet.	walking around	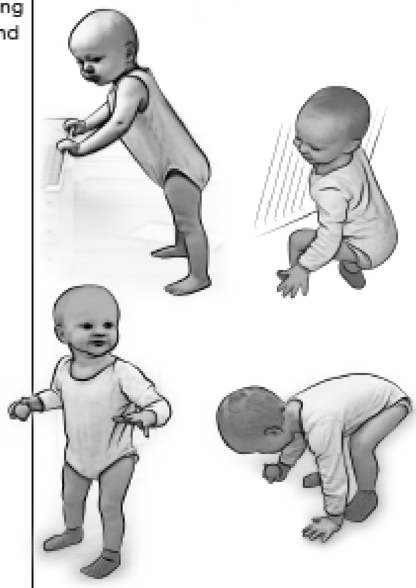
	☐ Encourage walking along the couch or table by replacing toys or making contact.		
	☐ Is your baby capable to transfer from a standing position to a sitting position? Try to capture this.		
	☐ If your baby is capable to stand or walk without support, try to capture this.		
	☐ Playing in a squatted position without support is the final position to film.		
